# Study on the Effect of N-Carbamylglutamate (NCG) on Reproductive Performance and Regulation Mechanism of Primary Lake Sheep

**DOI:** 10.3390/ani16030464

**Published:** 2026-02-02

**Authors:** Tianli Gao, Chunyang Li, Juanshan Zheng, Yingpai Zhaxi, Yuan Cai, Rongxin Zang, Huixia Liu, Yanmei Yang, Sai Li, Xiaodi Shi, Chen Huang

**Affiliations:** School of Life Science and Engineering, Northwest Minzu University, Lanzhou 730030, China; g3100662928@126.com (T.G.); lcy2625740914@163.com (C.L.); zhaxiyingpai@163.com (Y.Z.); rxzang2000@163.com (R.Z.); liuhuixia@aliyun.com (H.L.); yangyanmei@xbmu.edu.cn (Y.Y.); 13893186897@163.com (S.L.); shiody@163.com (X.S.); 18762305515@163.com (C.H.)

**Keywords:** Hu sheep, N-carbamylglutamate (NCG), transcriptomic sequencing, reproductive performance, fetal development

## Abstract

The aim of this study was to investigate the effects of dietary supplementation with 0.11% N-carbamylglutamate (NCG) during early pregnancy (0–90 days) on reproductive performance and fetal development, and to elucidate the underlying placental regulatory mechanisms in primiparous Hu sheep. Twenty-two 10-month-old sexually mature primiparous Hu sheep meeting the mating criteria were randomly assigned to two groups. The control group was fed a basal diet, while the NCG group received the basal diet supplemented with 0.11% NCG, with both feeding regimens maintained for 90 days. By measuring uterine and fetal growth indices, maternal plasma biochemical parameters, and amino acid levels, as well as assessing cotyledon indices and observing cotyledon morphology and histological structure, basic data related to placental function and fetal growth in pregnant ewes was collected. Combined with transcriptomic sequencing of maternal placental tissue, the mechanism by which NCG influences placental function and fetal growth and development in pregnant ewes was further investigated. The supplementation of NCG could increase the number of fetuses, total weight of fetuses, the number of corpus luteum and the ratio of fetuses to corpus luteum, but the difference was not significant (*p* > 0.05). The levels of plasma NO, inducible Nitric Oxide Synthase (iNOS) and several amino acids were significantly increased (*p* < 0.05). In ewes’ uteri, the average uterine weight, number of uterine glands, total cotyledon weight, and average weight per cotyledon were significantly increased (*p* < 0.05), whereas uterine mucosal thickness was markedly decreased. The Quantitative Real-time PCR (q-PCR) results for differentially expressed genes were consistent with those of transcriptomic analysis, showing significant changes in the expression levels of certain differentially expressed genes in maternal placental tissues. These changes regulated pathways such as vascular endothelial growth factor (VEGF), insulin-like growth factor (IGF), phosphatidylinositol 3-kinase–protein kinase B (PI3K–AKT) signaling pathways and Mitogen-Activated Protein Kinase (MAPK) pathway, which are involved in angiogenesis, energy supply and metabolism, and somatic growth and development. Dietary supplementation with NCG during early pregnancy can significantly improve the reproductive performance of primiparous Hu sheep, optimize the intrauterine environment and nutrient supply, and thereby facilitate pregnancy maintenance and fetal development. The underlying mechanism may involve promoting endogenous arginine synthesis in ewes, increasing plasma levels of NO, arginine, and certain amino acids, which collectively validate the positive effects of NCG on the reproductive performance and growth of Hu sheep during early pregnancy at the molecular level.

## Simple Summary

This study investigated the effects and molecular mechanisms of 0.11% N-carbamoylglutamic acid (NCG) supplementation during early pregnancy (0–90 days) on reproductive performance and fetal development in first-time calving Hu sheep. Twenty-two standard-compliant 10-month-old ewes were randomly divided into two groups: a control group receiving basal feed and an NCG group supplemented with 0.11%NCG for 90 consecutive days. By analyzing uterine/fetal indicators, maternal plasma biochemical parameters and amino acid levels, evaluating cotyledon characteristics, and performing placental transcriptome sequencing, the results indicated that early-pregnancy NCG supplementation may improve reproductive performance and fetal development through optimizing the uterine environment, promoting endogenous arginine synthesis, and increasing plasma nitric oxide (NO)/amino acid levels.

## 1. Introduction

China boasts a rich diversity of sheep and goat breeds, among which Hu sheep—a renowned multiparous breed [1]—have garnered significant attention due to their superior meat quality and high reproductive rate [2]. However, Hu sheep ewes are prone to issues such as intrauterine growth restriction (IUGR) owing to their small body size, high fecundity, and early mating age, which in turn impairs reproductive efficiency. Arginine (Arg), a functional amino acid, plays a pivotal role in animals. Studies have reported that L-arginine supplementation can improve semen quality and fertility after thawing in rams [3]. It serves as a precursor for the synthesis of proteins, nitric oxide (NO), polyamines, and other substances in the body [4]. As a signaling molecule, NO can enhance vascular dilation, thereby increasing blood flow [5], and exerts positive effects on somatic growth and development, intestinal immunity, and hormone regulation. Arginine also contributes positively to maternal placental development and maternal–fetal material exchange [6,7]. However, the widespread application of arginine is limited by its relatively high cost and susceptibility to degradation by rumen microorganisms in ruminant diets [8]. Thus, identifying a cost-effective and efficient arginine substitute is of particular importance.

N-carbamylglutamate (NCG), as an activator of carbamyl phosphate synthetase-1 (CPS-I), an essential cofactor for endogenous Arg synthesis [9], can promote endogenous Arg production. Compared with Arg, NCG is more cost-effective, less susceptible to degradation by digestive enzymes, and exerts significant effects even at low doses. Studies in monogastric animals have demonstrated that dietary NCG supplementation during pregnancy can reduce embryonic mortality [10], improve pregnancy rates and litter sizes [11], enhance placental vascular function [12], and facilitate the provision of more nutrients and oxygen by the mother to fetal tissues [13], thereby improving reproductive performance. In ruminants, studies have also shown that NCG can significantly increase the birth weight and growth performance of newborn lambs [14], exerting a positive impact on reproductive outcomes. In the preliminary trial, 0.05%, 0.08% and 0.11% NCG were screened, and the results showed that 0.11%NCG produced the best results, with 0.59 more lambs per litter and 0.68 live lambs per litter for the first group of ewes [15].

In order to investigate the effects of this formulation on reproductive performance and fetal development during early pregnancy (0–90 days), we administered 0.11% NCG to first-parity ewes. The comprehensive analysis included measuring uterine and fetal development indicators to reflect pregnancy outcomes and fetal growth status, plasma biochemical and amino acid profiles to reveal maternal metabolism and the state of NO and arginine pathways, hematoxylin-eosin (HE) staining to evaluate decidual structure and uterine gland/mucosal adaptation, and RNA-seq technology to identify differentially expressed genes (DEGs) in maternal placental tissues. These genes were enriched in pathways such as VEGF, IGF, PI3K-AKT, and MAPK, which are central to angiogenesis, nutrient transport, and fetal growth. Representative DEGs were also validated through q-PCR to confirm sequencing reliability and reveal the molecular pathways underlying these physiological changes.

## 2. Materials and Methods

### 2.1. Animals and Design

The experiment was conducted at Wanshan Cooperative in Qilihe District, Lanzhou City. Twenty-two ewes with normal estrus cycles and good health were selected for the trial. These twenty-two ewes were randomly divided into two groups: the control group received the basal diet (formulated according to NRC (2004) nutritional requirements for ewes weighing 40 kg during early pregnancy), and the experimental group received the basal diet supplemented with 0.11% NCG. The composition and nutritional levels of the experimental diet are shown in Table 1. The pre-feeding period lasted 10 days, followed by a 90-day feeding period. All ewes were synchronized through estrus induction and artificial insemination. The protocol was as follows: On day of 1, a CIDR device was inserted vaginally at 9:00 AM, with 3 mL of vitamin AD administered intramuscularly to each ewe. On day of 12, 250 units of PMSG were intramuscularly injected at 9:00 AM, followed by device removal at 6:00 PM. On day of 13, rams were introduced for estrus testing, with ewes standing and rams mounting as the estrus indicator, and estrus records were maintained. Fresh semen from healthy rams was collected daily, diluted with standard commercial semen diluent, and administered via the cervical canal in three consecutive doses, with a 12 h interval between each dose. The mating date was defined as day 0. The initial body weight of the ewes was 41.46 ± 4.72 kg. A total of 22 ewes underwent estrus synchronization and artificial insemination. Pregnancy diagnosis at day 35 showed that 20 out of 22 ewes were pregnant, yielding a pregnancy rate of 90.91%.

### 2.2. Measurement of Uterine and Fetal Indices

On day 90 of gestation, the ewes were euthanized by electrical stunning following venous blood collection. After excising the uterus, the following indices were measured and recorded: total cotyledon weight, withers height, fetal length, amniotic fluid volume, weights of various organs such as brain, liver, lung, kidney and stomach, average uterine weight, total litter weight, average individual cotyledon weight, fetal-to-corpus luteum ratio, and relative weights of fetal organs (relative to fetal body weight).

### 2.3. Blood Index Measurement

On day 90 of pregnancy, blood samples were collected from the jugular vein into Heparin sodium tubes. Samples were placed on ice and immediately centrifuged at 3500× *g* for 10 min at 4 °C. Serum was separated and stored at −80 °C until analysis. The NO, nitric oxide synthase (NOS), blood ammonia, estrogen (E2), and progesterone (PROG) were analyzed using ELISA kits from Nanjing Jiancheng (Nanjing, China), and the amino acid content was also measured by S-433D type automatic amino acid analyzerfrom Nanjing Jiancheng (Nanjing, China).

### 2.4. Cotyledon HE Staining Index Measurement

Cotyledons from each ewe’s placenta were randomly cut and fixed in 4% polyformaldehyde solution, embedded and cut into 5 micron slices. After fixation, the samples were transferred to 70% ethanol for long-term storage until subsequent treatment. Voracicular tissue of placenta was selected from the middle region of the pregnant uterine horn of each ewe, and four representative areas were selected for HE staining, including villous branches, mesenchyma, chorionic vessels and maternal cavity. The morphology of the placenta was observed by HE staining to ensure that both fetal and maternal parts were covered. The histological analysis was performed by measuring capillary density, thickness of uterine mucosa and number of uterine glands.

### 2.5. Transcriptome Sequencing Analysis

After slaughtering the sheep on day 90, maternal placental tissue from the uterus was quickly collected. Maternal placental tissue at the base of the small curvature of the uterine horn was cut with sterilized surgical scissors into 5–10 mm^3^ pieces, washed with PBS, placed in cryotubes, and stored in liquid nitrogen.

Three female sheep samples were randomly selected from both the NCG experimental group and the control group for transcriptome sequencing using the BGISEQ500 platform (BGI, Shenzhen, China). The 260/280 OD values of samples between 1.8 and 2.0 were selected for further analysis. Qualified RNA was used for library construction, and the transcriptome data were sequenced using BGI DNBSEQ platform (BGI, Shenzhen, China) for statistical analysis. The sequencing length was PE150, and the data were filtered by SOAPnuke 1.5.6 software. Raw reads were firstly processed using fastp and the low-quality reads were removed. The indexes based on Burrows–Wheeler transform and Ferragina–Manzini (FM) adopt two forms of genome-wide and local genome indexes to efficiently compare clean reads. The genome was compared using Hierarchical Indexing for Spliced Alignment of Transcripts (HISAT 2.2.1) [16] and Bowtie2 2.3.4.3 software [17]. Gene expression levels of each sample were calculated using RSEM v1.2.28 software. Differential expression analysis was performed between the NCG experimental group and the control group using DESeq2 1.40.2 software [18]. Differentially expressed genes (DEGs) were identified using the criteria of adjusted *p*-value (padj) < 0.05 and |log2 (fold change)| ≥ 1. Gene Ontology (GO) and Kyoto Encyclopedia of Genes and Genomes (KEGG) [19] pathway enrichment analysis of DEGs were, respectively, performed using R based on the hypergeometric distribution. Genes with *p*-value < 0.05 and fold changes (FC) > 1.5 were considered differentially expressed.

### 2.6. Real-Time Quantitative PCR

Quantitative real-time-PCR (RT-qPCR) was used to detect the mRNA levels of twelve genes. The same RNA samples were used for reverse transcription using a reverse transcriptase kit to synthesize first-strand cDNA. Primer Express Software v2.0 were used to design the primers and are detailed in Table 2. The housekeeping gene *GAPDH* was used as the standardized reference gene, and the relative expression level was calculated by 2^−ΔΔCt^.

### 2.7. Data Statistics and Analysis

Experimental data were statistically analyzed using SPSS 23.0, and intergroup differences were tested by *t*-test. Data are presented as means ± SEM. *p* < 0.05 was considered statistically significant. Mucosal thickness and uterine gland area and perimeter were calculated using Image-Pro plus 6.0. Transcripts with FC > 1.5 and *p* < 0.05 between the two groups were classified as DEGs.

## 3. Results

### 3.1. Effects of NCG Supplementation on Uterine and Fetal Index Development in Ewes During Early Pregnancy

As shown in Table 3, no significant differences were observed between the two groups in terms of average uterine weight, number of fetuses, birth weight, average fetal weight, number of corpora lutea, or fetal-corpus luteum ratio (*p* > 0.05). Notably, all indices except for average fetal weight exhibited an increasing trend relative to the control group, suggesting that NCG may promote fetal development in the uterus of ewes.

The data are presented as mean ± standard deviation, with a significance level of *p* < 0.05, The same applies to the table below.

As presented in Table 4, no significant differences (*p* > 0.05) were detected between the NCG-treated group and the control group with respect to fetal body dimensions or organ weights. These findings indicate that dietary supplementation with NCG during early pregnancy does not exert an influence on fetal body size parameters or internal organ weights.

### 3.2. Effects of Dietary NCG Supplementation on Blood Biochemical Indices and Amino Acid Content in Ewes During Early Pregnancy

As depicted in Table 5, the plasma levels of NO and inducible nitric oxide synthase (iNOS) in ewes from the NCG-treated group were significantly higher than those in the control group (*p* < 0.05), while progesterone levels in the treated group exhibited an increasing trend relative to the control group. No significant differences were observed between the two groups in terms of blood ammonia, estradiol, total nitric oxide synthase (TNOS), or endothelial nitric oxide synthase (eNOS) levels (*p* > 0.05). These results indicate that dietary supplementation with NCG during early pregnancy can significantly increase plasma concentrations of NO and iNOS in ewes.

As presented in Table 6, the plasma concentrations of glutamic acid, alanine, leucine, arginine, and proline were significantly higher in the NCG-treated group compared to the control group (*p* < 0.05). No significant differences were noted between the two groups with respect to the levels of other amino acids (*p* > 0.05). These findings suggest that dietary supplementation with NCG during early pregnancy can influence the plasma concentrations of certain amino acids in ewes.

### 3.3. Effects of NCG Supplementation on Cotyledon and HE Staining Indices in Ewes During Early Pregnancy

As illustrated in Table 7, amniotic fluid volume in the NCG-treated group exhibited an increasing trend relative to the control group. No significant difference was observed between the NCG-treated group and the control group in terms of cotyledon number (*p* > 0.05). However, both total cotyledon weight and average individual cotyledon weight in the NCG-treated group were significantly higher than those in the control group (*p* < 0.05). These results suggest that NCG can enhance cotyledon development.

As demonstrated in Table 8, the uterine mucosal thickness in the NCG-treated group was significantly lower than that in the control group (*p* < 0.05). No significant differences were found between the two groups in terms of capillary count, uterine gland area, or uterine gland perimeter (*p* > 0.05). However, the number of uterine glands in the NCG-treated group was significantly higher than that in the control group (*p* < 0.05).

### 3.4. Observation of Cotyledon Structure in Ewes on Day 90 of Pregnancy and Effects of NCG Supplementation on Its Development

**Figure 1:**
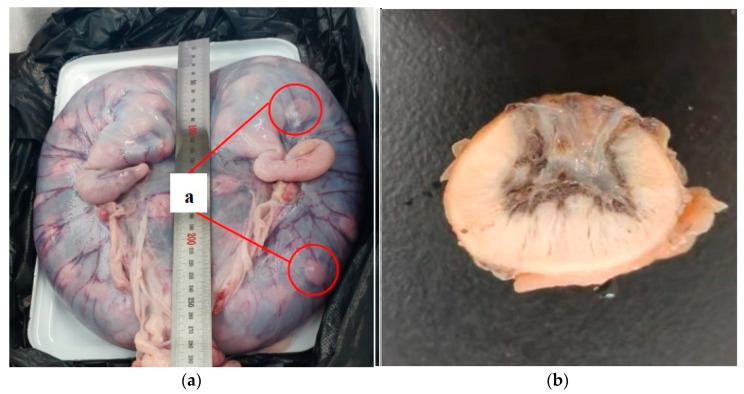
(**a**) Uterine diagram of hu sheep at 90 d of gestation, a, cotyledon; (**b**) Diagram of the anatomical characteristics of the Hu sheep.

The anatomical features of the placenta and uterus in 90 pregnant ewes are illustrated in Figure 1a. Hu sheep, as a multiparous breed, possess a cotyledonary placenta. The cotyledons are interconnected via an extensive network of uterine blood vessels, and maternal–fetal material exchange occurs through these cotyledon structures. The histological structure observation of cotyledon tissue in Hu Sheep in the 90th of pregnancy was observed as shown in Figure 1b.

**Figure 2:**
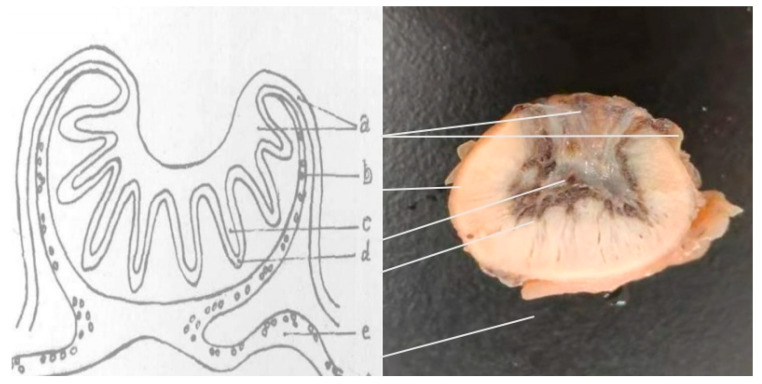
Model of cotyledon structure of sheep during pregnancy and corresponding anatomical characteristics of Hu sheep. a: Allantoic chorion. b: Uterine gland. c: Chorionic branch. d: Placental lacuna. e: Uterine mucosa.

As depicted in Figure 2, panel a shows the fetal placental component, while panels b and e represent the maternal placental components. Villi of the fetal allantochorion converge into villous clusters that are embedded within the maternal uterine mucosa. A key characteristic is that during pregnancy, cotyledons serve as the site of maternal–fetal material exchange, whereas the surfaces between cotyledons are generally smooth.

**Figure 3:**
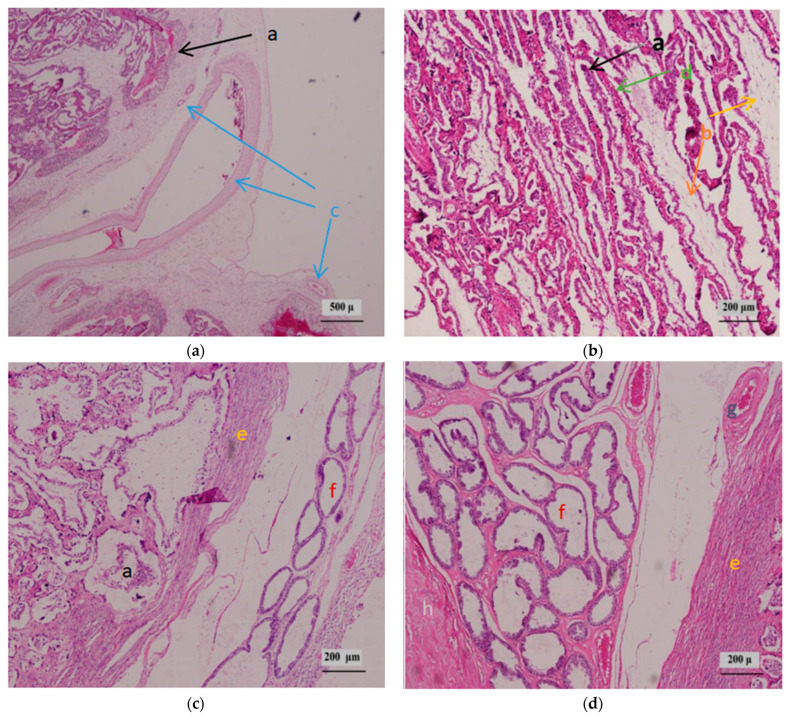
HE staining of sites (**a**–**d**). a, Villous branches. b, Mesenchyme c, Chorionic blood vessels. d, Maternal placental lacunae. e, Endometrium. f, Uterine glands. g, Uterine blood vessels. h, Myometrium.

As shown in Figure 1b, four different cotyledon tissue parts (a, b, c and d) were selected for HE staining observation, and the results of HE staining are shown in Figure 3.

Chorionic blood vessels: The cotyledons on day 90 contained a rich network of chorionic blood vessels, within which numerous red blood cells were visible. Vascular branches extended into the villous branches of the maternal placenta. Additionally, some underdeveloped blood vessels in the mesenchyme were small and remained in a closed state.

Mesenchyme: The mesenchyme, distributed between chorionic blood vessels and the chorionic epithelium, consists of cells with star-shaped cytoplasmic processes and columnar or elliptical nuclei. These cells are interconnected to form a network structure.

**Figure 4:**
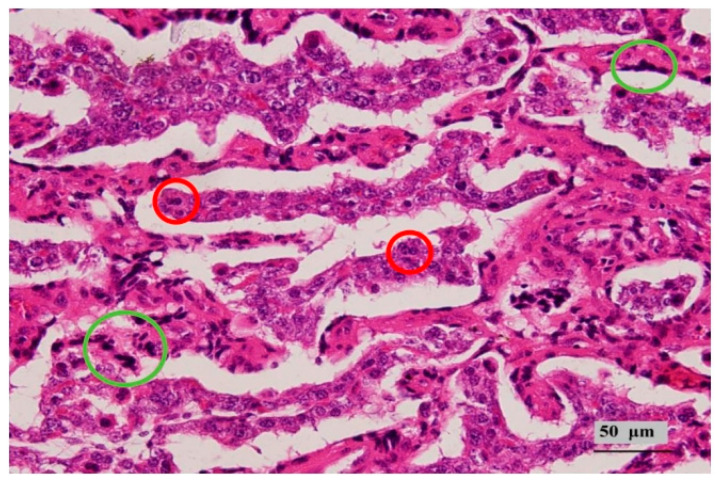
Syncytial and binuclear cells. The red circle is labeled as a double nucleus cell, and the green circle is labeled as a syncytium.

Chorionic epithelium: The chorionic epithelium is primarily composed of two cell types—binucleate cells and cuboidal cells—located on the surface of villous branches. In the cotyledonary chorionic epithelium of Hu sheep at 90 days of pregnancy, a large number of binucleate cells are widely distributed (Figure 4). Together, these cells form the surface structure of the fetal placenta, serving as a barrier between the fetus and the mother.

Uterine lacunar epithelium: At 90 days of pregnancy, the nuclei of the uterine lacunar epithelial cells are small, and the cells are flattened with obvious nuclear clustering (Figure 4). It has been suggested that this structure arises from the fusion of chorionic epithelial binucleate cells and uterine lacunar epithelial cells [19], and it plays a crucial role in maintaining pregnancy.

Uterine connective tissue: As branching of the uterine lacunar epithelium increases, the connective tissue becomes sparsely distributed and forms narrow structures. In the uterine mucosal layer adjacent to the uterine wall, connective tissue fibers are densely packed and neatly arranged, with numerous uterine glands distributed throughout.

Uterine blood vessels: In the cotyledons of Hu sheep at 90 days of pregnancy, uterine blood vessels are extensively distributed around the uterine mucosa and uterine glands, extending to the cup-like opening of the cotyledons. An abundance of uterine blood vessels is essential for ensuring normal fetal development.

### 3.5. Sequencing Quality Control and Comparison Analysis

The average data output per sample was 6.55 Gb. The average alignment rate of samples to the genome was 89.21%, with an average alignment rate of 63.90% to the gene set. Following quality control of the sequencing data, the average Q20 and Q30 values were 95.67% and 89.98%, respectively. These results demonstrate that the sequencing data are of high quality, high coverage, and reliability, making them suitable for subsequent differential analysis.

### 3.6. Quantitative Analysis of Gene Expression Levels

**Figure 5:**
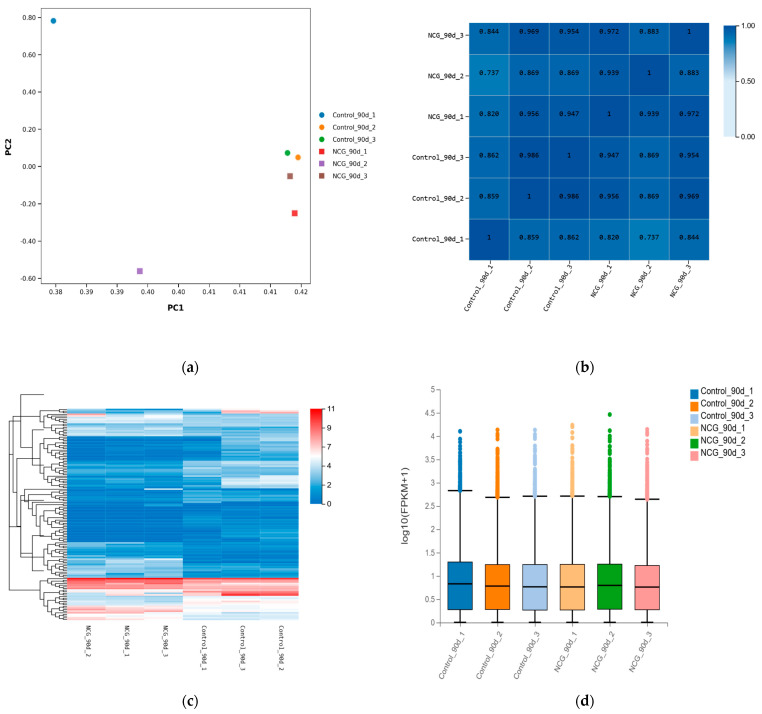
Integration of multidimensional statistical analysis and visualization technology. (**a**) PCA diagram, (**b**) correlation analysis, (**c**) heat map, (**d**) box plot.

Based on the alignment results, a total of 14,174 genes were detected in the control group, while 14,127 genes were identified in the NCG-treated group. The distribution of gene expression levels (FPKM/TPM) across different samples is presented in a boxplot (Figure 5d), which illustrates the median and interquartile range of expression values. As shown in Figure 5b, the intra-group correlation of samples in both the control group and NCG-treated group was analyzed. The results revealed that the correlation coefficient (R^2^) within each group exceeded 0.7 and approached 1, indicating good experimental reproducibility and validating the reliability of subsequent analyses.

### 3.7. Screening of Differentially Expressed Genes

**Figure 6:**
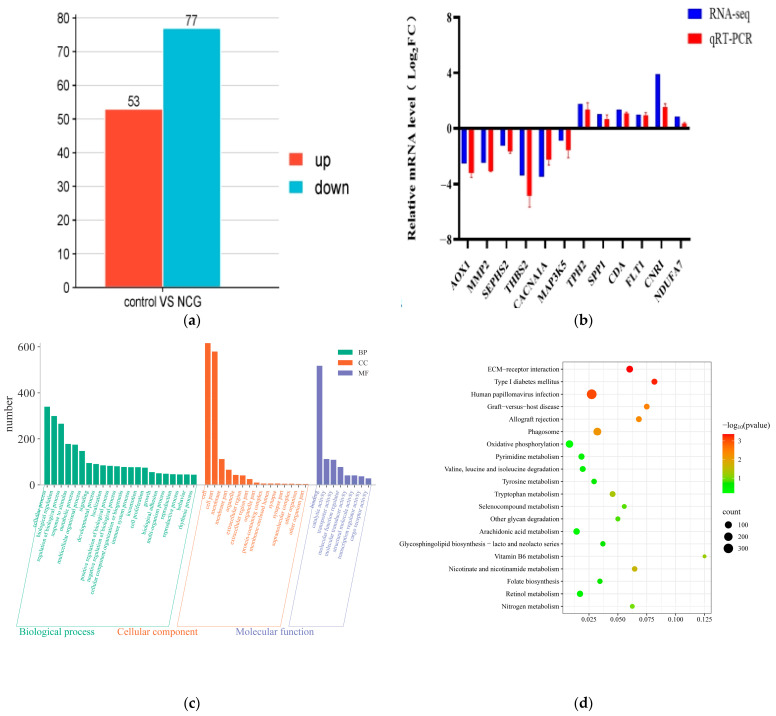
(**a**) Bar chart of differentially expressed genes between the two groups, (**b**) Comparison of qPCR and sequencing results of differential genes, (**c**) Gene Ontology (GO) enrichment classification of differentially expressed genes, (**d**) Kyoto Encyclopedia of Genes and Genomes (KEGG) pathway enrichment bubble diagram of differentially expressed genes.

In the visualization (Figure 6a), significantly upregulated genes are depicted in red, downregulated genes in blue, and non-significantly differentially expressed genes in gray. A total of 130 DEGs were identified, comprising 53 upregulated and 77 downregulated genes.

### 3.8. Gene Ontology Enrichment Analysis of Differentially Expressed Genes

As illustrated in Figure 6c, GO functional enrichment analysis was conducted on the DEGs identified between the control group and the NCG-treated group. A total of 45 significantly enriched GO terms were detected (*p* < 0.05), encompassing 22 biological processes (BPs), 15 cellular components (CCs), and 8 molecular functions (MFs). The BPs were primarily enriched in cellular processes, regulation of biological processes, responses to stimuli, and metabolic processes; the CCs were enriched in cells, cellular parts, membranes, and extracellular regions; and the MFs were enriched in binding, catalytic activity, transport activities, and molecular function regulators.

### 3.9. Kyoto Encyclopedia of Genes and Genomes Enrichment Analysis of Differentially Expressed Genes

As shown in Figure 6d, the KEGG pathway analysis listed the top 20 significantly enriched pathways associated with the identified DEGs, sorted by adjusted *p*-value (padj < 0.05). These pathways were found to include ECM-receptor interaction, oxidative phosphorylation, folate biosynthesis, and other processes related to organismal growth, development, nutrient supply, angiogenesis, vasodilation, fetal development, and signal transduction. Among the enriched pathways, the VEGF, Notch, Wnt/β-catenin, and PI3K-AKT signaling pathways are involved in processes such as cell growth and death, transport and catabolism, signal transduction, and amino acid metabolism.

### 3.10. Transcriptomic q-PCR Validation

To validate the RNA-seq results, 12 differentially expressed genes (DEGs) identified between the control group and the NCG treated group, including 6 upregulated and 6 downregulated genes, were randomly selected for q-PCR analysis. As shown in Figure 6b, the mRNA expression trends of all 12 validated genes were consistent with the RNA-seq data. Compared with the control group, the expression levels of *AOX1*, *MMP2*, *SEPHS2*, *THBS2*, *CACNA1A*, and *MAP3K5* were significantly downregulated in the NCG treated group, whereas those of *TPH2*, *SPP1*, *CDA*, *FLT1*, *CNR1*, and *NDUFA7* were significantly upregulated. These validation results confirm the reliability of the transcriptomic sequencing data, supporting that the identified DEGs accurately reflect the gene expression differences between the two groups.

## 4. Discussion

### 4.1. Effects of NCG Supplementation on Uterine and Fetal Development Indices in Ewes During Early Pregnancy

The results of this study showed that in the experiment when there was no significant change in fetal body size, relative organ weight and other indicators between the two groups, the NCG trial group was higher than the control group in uterine weight, fetal number, fetal weight per litter, fetal weight and other values. These findings suggest that dietary supplementation with NCG enhances maternal reproductive performance without exerting adverse effects on fetal development. The observed increase in uterine weight indicates more pronounced uterine growth, which may help reduce the incidence of IUGR during late gestation. Interestingly, although the average fetal weight in the NCG group did not differ significantly from that in the control group, a notable upward trend was detected in amniotic fluid volume. Nakano et al. [20] reported that this phenomenon may be attributed to NCG-induced alterations in amniotic fluid metabolism, which is consistent with previous reports by Wu [21]. Such metabolic modifications are likely to enhance nutrient supply in the maternal uterine environment, ensuring adequate nourishment for both the fetus and the mother, thereby improving embryonic survival rates.

### 4.2. Effects of NCG Supplementation on Blood Indices in Ewes During Early Pregnancy

Functional amino acids (FAAs) play critical roles in placental angiogenesis and development [22]. Among the most extensively studied FAAs involved in placental vascular regulation are arginine-family amino acids (including arginine, glutamic acid, proline, citrulline, and ornithine), leucine, and sulfur-containing amino acids. Notably, arginine exerts multifaceted biological functions, including promoting angiogenesis, enhancing nitrogen metabolism, stimulating lactation and growth, and improving reproductive performance in animals [23]. In this study, dietary supplementation with NCG significantly increased the concentrations of arginine, glutamic acid, proline and leucine in the plasma of ewes. Previous experimental results also demonstrated that the increase in amino acid concentrations did not adversely affect the fetus. This finding supports the hypothesis that NCG, as a structural analog of N-acetylglutamate (NAG), activates the key enzyme CPS-I, thereby enhancing endogenous Arg synthesis [24]. These results are consistent with previous work by Zhang [13], who reported that supplementing 20 g/d rumen-protected Arg (RP-Arg) and 5 g/d NCG under 50% feed restriction significantly increased plasma amino acid concentrations in Hu sheep. The observed elevation in amino acid levels may reflect synergistic interactions within the NCG metabolic pathway, which ultimately exert beneficial effects on the growth and physiological status of ewes.

The NO, hormones, and amino acids in maternal plasma play pivotal roles in regulating placental function and fetal development during pregnancy. The NOS catalyzes the production of NO, with iNOS—a key isoform—capable of generating substantial NO levels under immune stimulation. As a potent vasodilator, NO is critical for maintaining placental vascular function and optimizing nutrient delivery to the fetus [25]. Elevated NO levels may thus enhance maternal growth and reproductive performance by improving uterine blood flow and nutrient supply. In the present study, the NCG-supplemented group exhibited significantly higher plasma concentrations of NO and iNOS compared to the control group. These findings are consistent with previous reports by Wang [26], who observed a significant increase in plasma NO levels in lactating goats supplemented with 2 g/d NCG at 21 and 42 days postpartum. Furthermore, Zhang [27] demonstrated that NCG supplementation elevated plasma iNOS levels in IUGR lactating lambs, providing additional support for our results. Collectively, these data suggest that NCG enhances NO synthesis, potentially via iNOS activation, thereby improving placental vascular function and maternal–fetal nutrient exchange.

### 4.3. Effects of NCG Supplementation on Cotyledon and HE Staining Indices in Ewes During Early Pregnancy

Uterine glands are critical anatomical structures responsible for histotroph secretion and maternal–fetal material transport [28], playing an indispensable role in embryonic nutrition. Our experimental results showed that the number of uterine glands in the NCG-supplemented group was significantly greater than that in the control group. This observation is consistent with previous findings by Pramod [29], who reported that increased uterine gland density enhances nutrient secretory capacity, thereby supporting fetal development. The secretory function of uterine glands is mediated by glandular epithelial cells, with secretory capacity being directly proportional to the number of epithelial cells [30]. Although no significant differences were observed in the perimeter or cross-sectional area of individual glands between the two groups, the increased count of uterine glands in NCG-treated ewes suggests a corresponding increase in the total number of glandular epithelial cells. This histological adaptation likely facilitates enhanced nutrient exchange at the maternal–fetal interface.

Cattle and sheep both possess a cotyledonary placenta, yet their structural organization differs significantly [31]. Whereas bovine placentation follows a “fetus-enveloping-mother” pattern, ovine placentation exhibits an inverse “mother-enveloping-fetus” architecture, characterized by maternal caruncles forming bowl-shaped structures that encapsulate fetal cotyledons. Hematoxylin-eosin (HE) staining revealed that cotyledonary villous branches were highly abundant in Hu sheep at 90 days of gestation, with a complex internal structure. Maternal placental lacunae and villous branches were alternately nested, with distinct staining patterns: the maternal cotyledonary components appeared redder, while the fetal components were purple. Consistent with anatomical descriptions [32], the complete barrier structure of the cotyledonary tissue in Hu sheep comprises six layers from fetus to mother: chorionic blood vessels, mesenchyme, chorionic epithelium, uterine lacunar epithelium, uterine connective tissue, and uterine blood vessels. The first three layers form the fetal placenta, and the latter three constitute the maternal placenta. There is no direct blood contact between the mother and fetus; instead, they are connected via interlaced maternal lacunae and villous branches, which serve as the primary sites for nutrient supply, gas exchange, and metabolite excretion [33]. A large number of syncytiotrophoblasts are distributed within the maternal lacunae, a structure formed by the migration of fetal binucleate cells [34].

In this experiment, the average thickness of the uterine mucosa in the NCG-treated group was significantly lower than that in the control group. Cotyledons play a key role in embryonic development by expanding the effective maternal–fetal exchange area [35], which is determined by the number of fetal villous branches and their degree of embedding into the endometrium—with more branches and deeper embedding leading to an increased exchange area. Under the same conditions, a thinner maternal uterine mucosa allows for deeper chorionic embedding and more intensive material exchange within the cotyledons [36]. Given that elevated NO levels during pregnancy positively regulate extravillous trophoblast invasion [37], and that NCG supplementation increased NO content in the present study, it is suggested that NCG may enhance chorionic invasion into the maternal endometrium via NO-mediated pathways, thereby facilitating maternal–fetal communication.

### 4.4. Transcriptomic Sequencing and qPCR Validation of Maternal Placental Tissue

Through GO and KEGG enrichment analyses, differential expression analysis of upregulated and downregulated genes revealed that significantly upregulated genes were primarily enriched in pathways related to IGF binding, VEGF-A/B, PLGF, and VEGF receptor activity, while downregulated genes were enriched in pathways associated with heparin binding, Roundabout binding, and PGI synthase activity.

The VEGF is widely recognized as a key regulator of angiogenesis [38]. Its family members, including VEGF-A/B/C/D and PLGF [39], exhibit high homology [40]. As a potent endothelial growth factor, VEGF induces vasodilation and enhances blood flow by increasing NO production, while PLGF, commonly known as placental growth factor-1, acts as a strong enhancer of endothelial permeability. The VEGF shows high nutritional sensitivity. Previous studies have demonstrated that when nutrient intake transitions from adequate to inadequate, the expression of placental VEGF mRNA in cotyledons increases, which correlates with elevated maternal progesterone levels [41]. This aligns with our findings, indicating that the increased VEGF mRNA expression in cotyledons of NCG-supplemented pregnant Hu sheep may be directly mediated by progesterone. In placental lobules, nutrient restriction leads to increased *FLT1* mRNA expression [42], which is consistent with our observations in ewes. This is likely because *FLT1* functions as a receptor for VEGF [43]. The VEGF participates in neovascularization via the PI3K-AKT signaling pathway [44]. This pathway regulates fundamental cellular functions such as translation, proliferation, and growth arrest [44,45]. The KEGG pathway analysis further revealed close associations between the PI3K-AKT signaling pathway and VEGF. During angiogenesis and vascular network formation, blood flow within vessels induces shear stress on endothelial cells. This activates the KLF2 transcription factor, which responds to shear stress on the endothelial cell membrane, leading to increased expression of NOS regulatory proteins. This is supported by the elevated plasma NO and iNOS levels observed in our study, which ultimately regulate vascular endothelial growth factor receptor (VEGFR). Research has demonstrated that KLF2 regulation is mediated through the PI3K-AKT signaling pathway [46].

The *CNR1* gene regulates food intake and fat content in the hypothalamus [47]. *CNR1* and *CNR2* are members of the cannabinoid receptor (CNR) family. Schwartz’s team found that this gene is expressed in the central nervous system (including the hypothalamus) and is involved in appetite regulation [48]. Furthermore, *CNR1* can positively regulate the MAPK signaling pathway, which is crucial for cellular functions such as growth, differentiation, and apoptosis. It also promotes the early differentiation of skeletal progenitor cells into osteoblasts and accelerates bone mineralization [49]. Notably, the high expression of *CNR1* in the NCG group may be closely associated with the regulation of the MAPK pathway and cellular growth/differentiation.

Insulin-like growth factor (IGF) exerts biological activity by binding to cell surface receptors [50]. As a broad-spectrum growth promoter, IGF plays a crucial role in embryonic development [51]. The IGF-binding proteins (IGFBPs) exhibit diverse physiological functions [52], specifically, *IGFBP3* is involved in cell proliferation, differentiation, and apoptosis [53]. Gadd observed lower *IGFBP3* expression in uterine glands of adolescents with intrauterine growth restriction in high/medium dose groups, which aligns with our findings that NCG promotes uterine development in Hu sheep by enhancing IGFBP3 expression [54].

**Figure 7:**
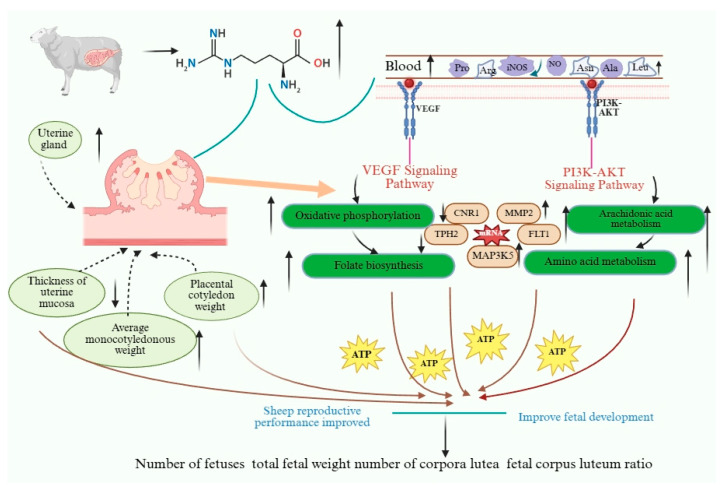
The addition of 0.11% in early-pregnancy diet regulated the reproductive performance and fetal development of ewes. ↑: Gene and pathway expression enhancement or promotion. ↓: Gene and pathway expression weakening or reduction.

Among the upregulated differentially expressed genes, *TPH2* was significantly increased in the NCG group. This gene catalyzes the conversion of L-tryptophan to 5-hydroxy-L-tryptophan (5-HT), thereby regulating levels of gonadotropin-releasing hormone (GnRH) and luteinizing hormone (LH) to support pregnancy maintenance and placental development. For downregulated differentially expressed genes, *MAP3K5* was significantly enriched in KEGG pathways, participating in MAPK signaling and endoplasmic reticulum protein processing. The *MAP3K5*, a mitogen-activated protein kinase kinase kinase, belongs to the *MAP3K5* family [55] and regulates intercellular junctions and the actin cytoskeleton [56]. In mammals, knockout of *MAP3K5* impairs brown adipose tissue function, accelerates energy expenditure, reduces fat accumulation, and induces metabolic disorders [57]. The upregulation of *CNR1* and downregulation of *MAP3K5* in uterine tissues are both associated with the MAPK signaling pathway. This suggests that NCG may influence fat deposition and energy metabolism in pregnant ruminants. Figure 7 shows the effect of dietary NCG on placental development in pregnant ewes during the first three months of gestation and the mechanism by which changes in VEGF and PI3K-AKT pathways improve reproductive performance.

## 5. Conclusions

This study demonstrated that dietary supplementation with 0.11% NCG in pregnant ewes (days 0–90 of gestation) significantly improved endometrial parameters in primiparous Hu sheep, including total endometrial weight, average individual cotyledon weight, mucosal thickness, and uterine gland count. The treatment enhanced reproductive performance by promoting endogenous arginine synthesis, increasing plasma levels of NO, arginine, and specific amino acids, and altering the expression patterns of genes such as *CNR1*, *TPH2*, *FLT1*, and *MAP3K5*, as well as signaling pathways related to angiogenesis, energy metabolism, and growth regulation. These changes optimized the uterine microenvironment and nutrient supply, thereby creating favorable conditions for maintaining pregnancy and supporting fetal development.

## Figures and Tables

**Table 1:** Composition and nutrient levels of diets (dry matter basis).

Items	Ingredient (%)
Corn	9.84
Wheat bran	2.53
Soybean meal	4.26
Salt	0.08
Premix	0.39
NaHCO_3_	0.08
CaHPO_4_	0.08
Alfalfa	44.86
Whole corn silage	37.88
Total	100
Nutrient levels	
DE(MJ·kg^−1^)	10.98
CP	16.08
Ca	1.30
TP	0.34

Premix provided per kg of diet: VA: 1637.21 IU, VD_3_: 388.84 IU, VE: 15.35 IU, niacin: 4.91 mg, Fe: 40.93 mg, Cu: 6.14 mg, Zn: 24.56 mg, Mn: 30.70 mg, I: 0.29 mg, Se: 0.10 mg, Co: 0.03 mg, Mg: 102.33 mg, Ca: 1023.26 mg, TP: 204.65 mg, NaCl: 1023.26 mg.

**Table 2:** Primer information.

Q-PCR Primers	The Sequence(5′-3′)	TM
*AOX1*-F	TGTGCTGGTGACTCACGGTG	59
*AOX1*-R	CCCGGAGGTGGATACTGGAC	
*TPH2*-F	TCTGCTGACGAAACACTGCG	59
*TPH2*-R	TCCCGAGGACTCAGGTACCC	
*MMP2*-F	CGGCATCTCTCAGATCCGTG	59
*MMP2*-R	CTTTTCCGGTAGCTCAGGCC	
*SPP1*-F	CCAAGGAGGAAAGCAAGCAT	59
*SPP1*-R	GGATTTTCAGGCGCTTGTCT	
*CDA*-F	GTTGCTGCTGTCCTGCCAAG	59
*CDA*-R	TTGCACCCGGAGAAGATCCT	
*SEPHS2*-F	CTCCTGCGTCATACCCCTGA	59
*SEPHS2*-R	CGATGCGCCCCATCATATAG	
*FLT1*-F	GCCTGCCGAGCTAGGAACAT	59
*FLT1*-R	GTGCGGTCACTGAGGTTTCG	
*THBS2*-F	CGACCTCTTCAGCCTCAGCA	59
*THBS2*-R	TAGTCAAAGCGGACGAAGCG	
*CACNA1A*-F	GTTTGAGAAAGATTGCCGCG	59
*CACNA1A*-R	GCCCACAGCACGTTGTCATA	
*MAP3K5*-F	GTCAGGTCCAGGTGGTGCTC	59
*MAP3K5*-R	CCGCCTTTCGGATGATACTG	
*CNR1*-F	CTCGGACTGCCTGCACAAAC	59
*CNR1*-R	AGACATGGTCACCTTGGCGA	
*NDUFA7*-F	GAGACTTGCAGGCGAAACTG	59
*NDUFA7*-R	GTTGGAGAGTCTGTGGCTGG	

**Table 3:** Effects of NCG on uterine weight, fetal index, and corpus luteum index.

Items	Control Group	NCG Group
Uterine average weight	3.54 ± 0.85	4.21 ± 0.62
Number of fetuses	1.80 ± 0.45	2.17 ± 0.41
litter fetal weight	918.99 ± 241.09	1084.21 ± 196.58
Fetal average weight	525.14 ± 62.38	500.40 ± 38.90
Corpus Luteum Count	2.20 ± 0.84	2.33 ± 0.52
Fetal luteal ratio	0.87 ± 0.18	0.94 ± 0.14

**Table 4:** Effects of NCG on fetal body size and organ weight.

Items	NCG Group	Control Group
body length	16.25 ± 0.76	16.80 ± 0.84
withers height	17.07 ± 1.22	16.97 ± 1.05
Brain weight ratio	27.34 ± 1.62	26.43 ± 2.45
Gastric weight ratio	14.61 ± 1.72	14.87 ± 1.32
Liver compared to	61.49 ± 2.89	61.64 ± 3.98
Kidney ratio	12.38 ± 1.26	12.58 ± 0.88
Lung size ratio	37.72 ± 3.04	38.11 ± 3.01

**Table 5:** Effects of NCG on plasma NO, NOS, ammonia, and hormone contents in ewes on day 90.

Items	NCG Group	Control Group
NO (µmol·L^−1^)	14.69 ± 2.29 ^a^	11.51 ± 2.09 ^b^
blood ammonia (µmol·L^−1^)	128.12 ± 9.68	120.43 ± 14.83
PROG (ng·mL^−1^)	5.22 ± 0.33	4.86 ± 0.25
E2 (ng·L^−1^)	33.22 ± 2.00	32.06 ± 1.96
TNOS (U·mL^−1^)	15.76 ± 1.46	16.80 ± 1.05
eNOS (U·mL^−1^)	12.62 ± 1.55	11.76 ± 1.90
iNOS (U·mL^−1^)	10.50 ± 1.06 ^a^	8.64 ± 0.90 ^b^

Note: Different lowercase letters in the column data indicate that the difference is significant (*p* < 0.05), and the same letters indicate that the difference is not significant (*p* > 0.05).

**Table 6:** Effects of NCG on plasma amino acid content in ewes on day 90.

Items (µmol/L)	NCG Group	Control Group
Asp	12.44 ± 9.17	7.27 ± 1.27
Thr	62.68 ± 28.46	39.70 ± 7.97
Ser	87.21 ± 14.78	75.92 ± 25.47
Asn	65.70 ± 14.94	50.05 ± 12.06
Glu	149.82 ± 21.50 ^a^	119.96 ± 19.64 ^b^
Gly	613.62 ± 100.39	522.81 ± 89.83
Ala	356.55 ± 33.92 ^a^	308.93 ± 13.01 ^b^
Cit	184.20 ± 54.09	195.76 ± 38.79
Val	146.09 ± 41.23	141.04 ± 16.47
Met	45.59 ± 5.20	41.85 ± 2.23
Ile	101.85 ± 10.72	87.57 ± 13.15
Leu	143.93 ± 14.73 ^a^	122.05 ± 9.98 ^b^
Tyr	74.85 ± 9.30	70.17 ± 14.31
Phe	30.68 ± 5.91	29.33 ± 3.81
His	59.68 ± 11.42	51.16 ± 6.80
Trp	600.05 ± 49.37	596.12 ± 60.53
Orn	70.24 ± 23.49	53.52 ± 11.27
Lys	285.61 ± 42.33	272.05 ± 34.54
Arg	207.81 ± 27.21 ^a^	167.55 ± 29.46 ^b^
Pro	146.96 ± 19.18 ^a^	123.90 ± 7.06 ^b^
Asp	12.44 ± 9.17	7.27 ± 1.27

Note: Different lowercase letters in the column data indicate that the difference is significant (*p* < 0.05), and the same letters indicate that the difference is not significant (*p* > 0.05).

**Table 7:** Effects of NCG on amniotic fluid volume and cotyledon indices.

Items	NCG Group	Control Group
Amniotic fluid volume	566.42 ± 40.76	526.40 ± 35.96
Number of cotyledons	88.67 ± 14.09	88.00 ± 15.43
total weight of cotyledons	777.50 ± 79.98 ^a^	641.00 ± 74.53 ^b^
average weight of monocotyledons	8.84 ± 0.77 ^a^	7.38 ± 0.85 ^b^

Note: Different lowercase letters in the column data indicate that the difference is significant (*p* < 0.05), and the same letters indicate that the difference is not significant (*p* > 0.05).

**Table 8:** Effects of NCG on mucosal thickness, capillary, and uterine gland indices in cotyledon.

Items	NCG Group	Control Group
thickness of the endometrial lining	256.59 ± 94.59 ^b^	422.76 ± 116.18 ^a^
Number of capillaries	11.17 ± 1.72	10.50 ± 1.87
Number of uterine adenoma	26.33 ± 3.06 ^a^	19.00 ± 2.65 ^b^
area of uterine glands	47,283.32 ± 5694.17	43,454.43 ± 5281.84
perimeter of uterine glands	923.11 ± 94.35	955.25 ± 97.80

Note: Different lowercase letters in the column data indicate that the difference is significant (*p* < 0.05), and the same letters indicate that the difference is not significant (*p* > 0.05).

## Data Availability

The data requested by your journal has been submitted to the GSA database at the National Center for Biotechnology Information (NCBI), with the login number PRJNA1302455.
